# Cardiometabolic risk in people under 40 years with severe mental illness: reading between the guidelines

**DOI:** 10.1007/s11096-023-01600-1

**Published:** 2023-05-22

**Authors:** Aoife Carolan, Caroline Hynes, Stephen McWilliams, Cristín Ryan, Judith Strawbridge, Dolores Keating

**Affiliations:** 1 Saint John of God Hospital, Stillorgan, Co. Dublin, Ireland; 2 School of Pharmacy and Biomolecular Science, Royal College of Surgeons Ireland, 123, St Stephen’s Green, Dublin 2, Ireland; 3 School of Medicine and Medical Sciences, University College Dublin, Belfield, Dublin 4, Ireland; 4 School of Pharmacy and Pharmaceutical Sciences, Trinity College , Dublin 2, Ireland

**Keywords:** Cardiometabolic risk, Intervention, Multimorbidity, Risk assessment, Severe mental illness

## Abstract

People with severe mental illness (SMI) have a shorter life expectancy than the rest of the population. Multimorbidity and poorer physical health contribute to this health inequality. Cardiometabolic multimorbidity confers a significant mortality risk in this population. Multimorbidity is not restricted to older people and people with SMI present with multimorbidity earlier in life. Despite this, most screening, prevention and treatment strategies target older people. People under 40 years with SMI are underserved by current guidelines for cardiovascular risk assessment and reduction. Research is needed to develop and implement interventions to reduce cardiometabolic risk in this population.

People with severe mental illness (SMI), including schizophrenia, schizoaffective disorder, bipolar illness and severe affective disorder have a life expectancy which is up to 30 years shorter than those without SMI [1–4]. Multimorbidity (the presence of two or more long-term health conditions with no one condition being considered as the index) and poorer physical health contribute significantly to this health inequality [5, 6].

Certain morbidities cluster together [5, 7]. Identifying and understanding these clusters is becoming increasingly important in our approach to multimorbidity [8]. Cardiometabolic multimorbidity is defined as the co-existence of two or more of three cardiometabolic disorders: hypertension, diabetes mellitus and cardiovascular disease (CVD) [9]. A cluster analysis of data from the United Kingdom (UK) showed that diabetes and CVD were the largest group of multimorbidity conditions [5]. Similarly, in a study investigating multimorbidity patterns in older people, cardiovascular and metabolic disorders were the most prevalent combination, affecting 30% of females and 39% of males [10].

Mortality risk from cardiometabolic multimorbidity is significant [5]. The Emerging Risk Factor Collaboration estimated that at 60 years of age, those with cardiometabolic multimorbidity had an estimated 12 years of reduced life expectancy compared to those without cardiometabolic multimorbidity [11].

Multimorbidity is not restricted to older people (i.e. ≥ 65 years). Those with SMI present with multimorbidity earlier in life than those without SMI [8, 12]. An accumulated prevalence analysis of UK primary care data observed that physical health conditions cluster similarly in people with and without SMI, although patients with SMI had a higher burden of multimorbidity, particularly in younger age groups [12]. A Scottish study by Barnett et al. in 2012 found that although the prevalence of multimorbidity is much higher in older people than in those under 65 years of age, more than half of people with multimorbidity and nearly two-thirds with physical-mental health comorbidity were younger than 65 years [13]. The impact of multimorbidity on survival may vary significantly across different age groups with relative mortality risk being higher among younger male adults with multimorbidity [14].

To date, interventions to screen, prevent and treat multimorbidity including cardiometabolic multimorbidity, have mostly targeted older adults. Interventions for younger people are lacking [12, 14].

CVD confers the highest risk of mortality in cardiometabolic multimorbidity [9]. Interventions to assess and reduce cardiovascular risk are supported by national and international guidance but the implementation of these guidelines is poor, not least amongst those with SMI. UK national audit data suggests that less than half of people with a QRISK score of 10% of more are on lipid-lowering therapy [15]. The National Clinical Audit of Psychosis for Early Intervention Services in Ireland includes a standard that people should receive a physical health review annually which includes smoking status; alcohol intake; substance misuse; BMI; blood pressure; glucose and cholesterol. In 2020/2021, 33% of patients met the standard for lipid monitoring and 27% met the standard for all seven parameters [16].

The rising prevalence of multimorbidity and associated complexity of disease and polypharmacy presents challenges for those who treat SMI. Fragmented health care service provision, increased specialisation of health professionals and diagnostic overshadowing means that patients with SMI have multiple isolated visits to different healthcare settings and are prescribed different medicines by different health professionals. Whitty et al. 2020 asked health professionals to rise to this challenge and warned that unless we react to this problem, people with multimorbidity will be disadvantaged. Identifying multimorbidity clusters and designing systems to better manage coexisting physical and mental health problems should be a priority [8].

Screening and assessment tools can potentially help improve physical outcomes and reduce this inequality [17, 18]. A medicines optimisation tool, OPTIMISE, was developed and validated to assist clinicians working in psychiatry to protect the overall physical health of people with SMI. OPTIMISE prompts the regular assessment of CV risk in adults with SMI over 40 years using a validated risk assessment tool e.g. SCORE, QRISK2, QRISK3, ensuring that at a minimum, people with SMI are screened for CVD in equal measure with the rest of the population [18]. In applying this tool in practice, the authors identified that adults under 40 years with SMI are underserved by the current guidelines.

As they stand, current guidelines for the assessment and reduction of CV risk do not provide clear guidance for assessing and managing CV risk in adults under 40 years of age. The National Institute for Health and Care Excellence (NICE) clinical guideline (CG 181) on CVD: risk assessment and reduction including lipid medication states that people older than 40 should have their CVD risk estimate reviewed on an ongoing basis [15]. Similarly, European guidelines state that systematic CVD risk assessment in the general population (adult men > 40 and women > 50 years of age) with no known CV risk factors appears not to be cost-effective in reducing subsequent vascular events and premature death, at least in short-term follow-up [19]. This means that even when healthcare systems adhere to the standards for monitoring and intervention to reduce CV risk, younger people with SMI may be missed.

The most recent iteration of NICE guidance for CV risk assessment, currently in draft format, allows for greater clinical judgment when intervening to reduce CV risk. This draft guidance allows for a more individualised approach and suggests that statins can be considered as an option for people with 10-year risk scores that are less than 10%. It is suggested that this would be appropriate if a person has risk factors which are not covered by QRISK3 [20]. This is a welcome change but more research is needed in developing and implementing interventions to reduce CV risk in this high-risk population.

In summary, people with SMI are more likely to experience multimorbidity earlier in life that the rest of the population. There is evidence of increased prevalence of cardiometabolic multimorbidity in people under 40 years with SMI. Cardiometabolic multimorbidity is strongly associated with higher mortality risk yet national and international guidelines to assess and reduce CV risk are targeted at those aged 40 or over. Health systems currently struggle to implement CV risk assessment recommendations to the general population not least those with SMI. Research is needed to develop and implement interventions to reduce cardiometabolic risk in adults under 40 with SMI as current guidelines do not yet recommend routine assessment and intervention.
